# A Novel System for Quasi-Continuous THz Signal Transmission and Reception

**DOI:** 10.3390/s22124448

**Published:** 2022-06-12

**Authors:** Andrej Sarjaš, Blaž Pongrac, Dušan Gleich

**Affiliations:** Faculty of Electrical Engineering and Computer Science, University of Maribor, Koroška Cesta 46, 2000 Maribor, Slovenia; blaz.pongrac1@um.si (B.P.); dusan.gleich@um.si (D.G.)

**Keywords:** quasi-continuous, terahertz, photoconductive antenna, wave emittance, wave detection

## Abstract

This paper presents a novel system for generating and receiving quasi-continuous (QC) TeraHertz (THz) waves. A system design and theoretical foundation for QC-THz signal generation are presented. The proposed QC-THz system consists of commercially available photo-conductive antennas used for transmission and reception of THz waves and a custom-designed QC optical signal generator, which is based on a fast optical frequency sweep of a single telecom distributed-feedback laser diode and unbalanced optical fiber Michelson interferometer used for a high-frequency modulation. The theoretical model for the proposed system is presented and experimentally evaluated. The experimental results were compared to the state-of-the-art continuous-wave THz system. The comparison between the continuous-wave THz system and the proposed QC-THz system showed the ability to transmit and receive QC-THz waves up to 300 GHz. The upper-frequency limit is bounded by the length of the used Michelson interferometer. The presented design of THz signal generation has a potential for industrial application because it is cost-efficient and can be built using commercially available components.

## 1. Introduction

The development of devices and components using a frequency band between 0.1 and 10 THz [1] has increased rapidly over the past two decades. The electronic components for managing the THz band have become commercially available. Most of the research in the field of THz bands has concentrated on the excitation and probing principles. In recent decades, the first pilot applications appeared in different scientific and industrial areas. Nowadays, many different applications in the field of THz devices can be found for material scanning and food inspection [2,3], imaging [4], tissue analysis [5], security scanning [6], etc. Unlike the other inspection techniques based on X-rays and infrared radiation, the THz represent non-ionizing waves with low quantum energy, which cannot damage the inspected material permanently, and has good penetration ability. The THz technology is used mainly for non-destructive inspection and condition monitoring. The last emerging development and successfully applied industrial applications of the marked research showed that THz scanning is one of the top 50 emerging technologies [7].

Nowadays, there are only a few available and compact THz devices for use in industrial applications. Most of the THz applications use time or frequency domain spectroscopy, THz imaging, dilatation, or proximity detection with fixed or narrowed THz bands. The THz spectroscopy offers broad and unique applications in different areas [8,9,10]. The time-domain spectroscopy (TDS) usually uses short, femto-second laser pulses; meanwhile, the frequency domain spectroscopy (FDS) uses continuous waves (CW) to stimulate the photoconductive antenna. Therefore, TDS is the most common approach in THz spectroscopy. The main drawbacks of the TDS system are the complex and expensive components, such as a femtosecond laser (FSL) source, which mainly present barriers to industrial applications. The THz wave excitation in an FDS system is performed by using two continuous laser sources and photo mixing, which results in a monochromatic THz wave through the photoconductive antennas (PCAs) [11]. The produced wave has high temporal coherence; therefore, the measured data processing is more complex and uncertain. Unlike the TDS, the TDF components are more affordable. The use of PCAs provides an opportunity to develop small and compact THz wave sources and THz wave detectors. The extensive research of the decade confirms that the CW systems based on PCAs were the most reliable and robust. They were established as the primary methods for generating and detecting THz waves utilizing the PCAs. The only weaknesses of the CW systems are the complexity and price regarding the established industrial price expectations [12]. Researchers attempted to develop cost-efficient and less complex THz systems over the years, such as the proposed system in [13], where a single bimodal laser diode was utilized to generate an optical signal and THz signal. The principle in [13] was that a bimodal laser diode would emit light with two constantly spaced spectral lines. The emitted light from a bimodal laser diode is mixed in a waveguide, resulting in a modulated quasi-continuous wave (QCW) with the THz bandwidth.

In this paper, we proposed a quasi-continuous wave (QCW-THz) system that utilizes an optical signal generator (OSG), presented in a preliminary work [14], with the PCA emitter and PCA detector. The proposed OSG is based on commonly available and low-cost electronic components used widely in telecommunications. Such a cost-efficient optical device is used as an optical exciter of THz waves and can be utilized effectively in FDS and radar systems. The proposed OSG utilizes a rapid temperature cycling of a single Distributed-Feedback (DFB) laser diode and the unbalanced optical fiber Michelson interferometer for high-frequency modulation. Rapid temperature cycling of the DFB laser diode causes a frequency shift of the emitted light. A linear and fast optical frequency sweep (OFS) can be achieved using the appropriately designed DFB laser diode’s current driving the waveform. The predetermined DFB laser diode’s non-linear characteristics linearize the relation between driving current and temperature in the DFB laser diode’s active area, allowing simplified signal form generation. The current driving the waveforms determines the OFS, and in contrast, the optical signal’s high-frequency modulation. The waveforms for linear OFS and non-linear OFS were designed in the preliminary work. The linear OFS was used for a fixed frequency source, whereas the non-linear OFS was used for chirped signals. The emitted light from the DFB laser diode is modulated within the unbalanced optical fiber Michelson interferometer. In the preliminary work [14], a modulation frequency up to 300 GHz was achieved and confirmed with the measurement. Regarding the analysis of the current waveform, structure of the DFB laser diode and high-frequency modulation using the optical fiber interferometer, the theoretical limit of the system was set to 1.25 THz. The proposed system can tune different optical waveforms within a user-selected frequency span.

The proposed QCW-THz system is based on the CW-THz system’s working principle [11]. The QCW optical signal, generated with the proposed OSG, is composed of short bursts of CW signals with THz carrier frequency and is limited by the windowing function. The proposed QCW-THz system’s theoretical model was obtained according to the CW-THz properties. The developed QCW-THz theoretical model shows that the observed peak envelope values of the detector PCAs induced photocurrent represent the detected QCW signal with the THz carrier frequency. Therefore, the THz wave’s propagation information can be extracted from the induced current’s peak envelope value in the PCA receiver. The proposed QCW-THz system is more versatile than the current CW-THz system. The ability to tune the frequency inside the current waveform’s repetition rate and generate different optical waveforms is a significant advantage of the proposed device.

The theoretical model for the proposed QCW-THz system is derived in this paper. After the theoretical analysis, two unbalanced optical fiber Michelson interferometers are presented for the target frequencies of 1.45 and 289.33 GHz. The first interferometer, with a target frequency of 1.45 GHz, was used for OSG validation. The second interferometer, with a target frequency of 298.33 GHz, was used for the proposed QCW-THz system’s validation. The maximal peak-induced photocurrent value on the PCA detector was assured with PCA calibration and performed with a CW-THz system.

The proposed QCW-THz system was evaluated and compared to the CW-THz system TeraScan 1550 from Toptica Photonics. The PCA detector responds to inbound optical signals with an induced photocurrent. Two different experiments were performed to determine the induced photocurrent caused by the inbound THz waves in the PCA detector. An analysis of the PCA detector’s response with an unobstructed THz beam path between the PCA emitter and detector was observed in the first experiment. Furthermore, the THz beam path between the PCA emitter and detector was blocked in the second experiment; therefore, only the bias-induced photocurrent was detected, caused by an inbound optical signal. The difference in the observed maximal peak envelope currents from the first and second experiments represents the induced photocurrent in the PCA detector caused by the inbound THz waves.

The experimental results confirmed that the maximal achieved frequency of the proposed QCW-THz system was 289.33 GHz with the induced photocurrent of 46 nA. In contrast to the CW-THz at the same frequency and induced photocurrent of 51 nA, the proposed QCW-THz system is indicated as a cost-effective generator of THz waves. We showed that the performance between QCW-THz and CW-THz is very similar.

## 2. Theoretical Modeling of a QCW-THz System

**Figure 1:**
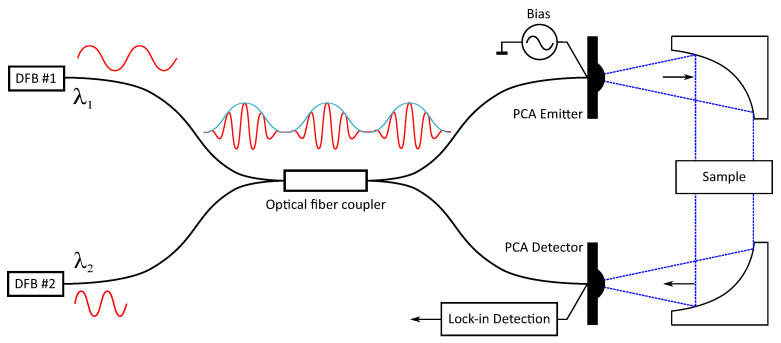
The basic structure of the CW-THz system.

This section presents a theoretical model of the proposed QCW-THz, based on the work presented in [11]. The basic principle of the CW-THz system is presented in Figure 1. The CW-THz consists of three major components: (1) an optical signal generator (OSG), (2) a THz wave transmitter and (3) a THz wave receiver. The OSG is used for generating an optical signal with modulation frequency in the THz band. The OSG uses the beating of two optical signals on different wavelengths to generate a beating optical signal and a pair of PCAs for emitting and detecting THz waves, as shown in Figure 1. The tunable DFB laser diodes are used as optical sources. The emitted THz beam is collimated with collimation mirrors into the PCA detector. The THz transmitter utilizes a PCA emitter and the PCAs bias voltage generator, while the THz receiver utilizes a PCA detector and a lock-in amplifier.

**Figure 2:**
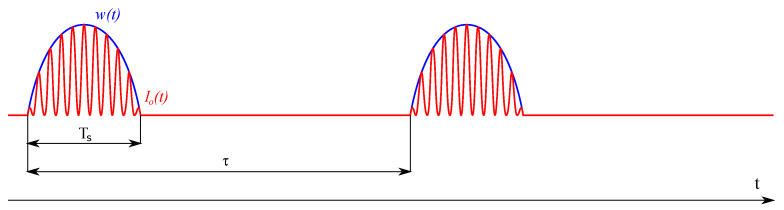
Proposed QCW signal waveform.

The principle of the proposed QCW-THz signal is shown in Figure 2. The idea is to generate a pulse-based signal.

The THz transmitter and THz receiver were modeled theoretically, as presented in [15]. The inbound QCW optical signal intensity at the PCA emitter’s and PCA detector’s gap is modeled as

(1) $$I_{o}\left(t\right)=w\left(t\right)\cdot I\cdot \cos\left(2\pi ft\right)$$

where *I* is the initial optical intensities modulated with the THz frequency *f*, and w(t) is a normalized windowing function. The biased PCA emitter acts as a capacitor with the charge $E_{Bias}$ if no light is present [16]. When the gap in the PCA emitter is illuminated by the optical signal, the photocarriers generate a gap field with a charge $E_{G}$. If the photoconductive material’s carrier lifetime is much shorter than the period 1/f, $E_{G}$ follows the waveform of $I_{o}$. The biased field $E_{Bias}$ pushes apart the generated photocarriers, which induces a photocurrent. The photocurrent drives a dipole antenna, and the THz wave is established. The THz far-field $E_{THz}$ can be estimated as

(2) $$E_{THz}\left(r,t\right)=w\left(t\right)\cdot E_{0}\left(r\right)\cos\left(2\pi f\cdot t+\phi \right)$$

where *r* is the distance from the source, and $E_{0}\left(r\right)$ is the distance-dependable THz far-field peak value.

Photocarriers are induced when the optical signal lights the detector PCAs gap. The induced photocarriers form the charge in the PCAs gap. The inbound THz waves then push apart the induced photocarriers with the induced voltage $V_{THz}$, estimated as

(3) $$V_{THz}=w\left(t\right)\cdot V_{0}\cdot \cos\left(2\pi f\cdot t+\phi_{THz}\right)$$

where $V_{0}=E_{0}\cdot l_{a}$ is the peak induced voltage with parameters $E_{0}$ and $l_{a}$ the peak inbound THz field and the length of the detector antenna, respectively. The separation of photo carriers induces a photocurrent $J_{p}$, which can be estimated as

(4) $$J_{p}\left(t\right)=V_{THz}\left(t\right)\cdot G\left(t\right)$$

(5) $$J_{p}\left(t\right)=w\left(t\right)\cdot J_{0}\left(\cos\left(\phi_{THz}-\phi_{o}\right)+\cos\left(2\pi f+\phi_{THz}+\phi_{o}\right)\right)$$

where G(t) is the light intensity-dependent conductivity of the detector PCAs gap and $J_{0}=V_{0}G_{0}$ is the peak-induced photocurrent. Only the offset or bias component can be observed due to the induced photocurrent’s high frequency. The bias component of the induced photocurrent in the PCA detector can be presented as

(6) $$J_{p}=w\left(t\right)\cdot J_{0}\cos\left(\Delta \phi \right)$$

where $\Delta \phi =\phi_{THz}-\phi_{o}$ represents the phase difference between the optical signal’s phase $\phi_{o}$ and THz wave’s phase $\phi_{THz}$. If the optical fibers leading to the PCA emitter and detector are of the same length, the phase difference Δϕ depends on the distance between antennas and medium propagation dispersion. If the distance between the PCA emitter and detector does not match the phase length of the propagation wave $L\neq N\cdot \lambda_{p}$, where *N* is the positive integer multiplication factor, the propagated wave’s phase shift of

(7) $$\Delta \phi_{L}=2\pi \cdot \frac{L}{N\cdot \lambda }$$

The dispersion or change in propagation speed will cause the propagated wave’s phase shift. The phase difference $\Delta \phi_{D}$ due to the dispersion in a medium can be estimated as

(8) $$\Delta \phi_{D}=\frac{2\cdot \pi }{\lambda_{p}}\cdot \left(n_{2}-n_{1}\right)\cdot D=\frac{2\cdot \pi \cdot f}{c}\cdot \left(n_{2}-n_{1}\right)\cdot D$$

where $n_{1}\ll n_{2}$, and D is the thickness of the propagation medium.

The transmitted THz wave is also impacted by the absorption in the propagation medium and observed sample. In transmission-based systems, absorption *A* can be estimated with transmittance. The transmittance *T* is defined as the propagated wave’s part detected after propagation through the medium and can be estimated as $T=10^{-A}$. In practice, the transmittance is estimated as the ratio given as

(9) $$T=\frac{J_{s}}{J_{r}}$$

where $J_{s}$ is the observed induced photocurrent in the PCA detector with a sample placed between the PCA antennas, and $J_{r}$ is the reference-induced photocurrent in the PCA detector with an unobstructed THz beam path.

The phase shift due to the distance between the PCA antennas, dispersion and absorption in the medium is added to Equation (6) and is

(10) $$J_{p}\left(t\right)=w\left(t\right)\cdot T\cdot J_{0}\cdot \cos\left(\Delta \phi +\Delta \phi_{D}+\Delta \phi_{L}\right)$$

In Equation (10), the only time-dependent variable is the normalized windowing function w(t). It should be emphasized that the peak envelope of induced photocurrent $J_{p}\left(t\right)$ in the PCA detector has the same waveform as the normalized windowing function w(t). The peak value of the measured photocurrent in window w(t) withholds the information about the observed medium and can be extracted similarly as in the CW system. The detected photocurrent depends strongly on the phase difference between the optical signal and inbound THz wave. The phase difference, which originates from the PCAs positioning, and dispersion in the observed medium, and their correlation, is presented in the derived model in Equation (10).

Maxwell equations can describe the THz wave propagation through the sample accurately [1]. The frequency-dependent absorption and dispersion in the observed medium will impact the amplitude and phase of the detected THz wave, as modeled in Equation (10). Absorption affects the amplitude of the propagated wave and is related to the permittivity of the medium ϵ, and the refractive index of the medium *n* with the Kramer–Kronig relation [17]. In conducting materials, such as metal, the dielectric constant is considered infinite, and the absorption is considered infinite—metals are opaque to THz waves due to their high absorption index. If the THz beam path in the proposed QCW-THz system is blocked with metal, the induced photocurrent in the PCA detector is equal to 0, and the transmittance is T=0. Furthermore, if the THz beam path in the proposed QCW-THz system is unobstructed, the induced photocurrent is maximal, and the transmittance is T=1. The difference between the detected induced photocurrent at T=0 and T=1 represents the induced photocurrent as a result of the inbound THz field.

The proposed theoretical model in Equation (10) does not take into account the optical bias. Optical bias, or bias photocurrent, is induced while the PCA detector is lit. In CW systems, it is usually filtered out by a Trans-impedance Amplifier (TIA) or Lock-in Amplifier (LIA) since the optical bias could potentially impact the LIA’s signal-to-noise ratio (SNR). In the proposed QCW system, the optical bias is added in Equation (10). An induced photocurrent with optical bias term is equal to:

(11) $$\begin{array}{c} J_{p}\left(t\right)=w\left(t\right)\cdot \left(J_{b}+T\cdot J_{0}\cdot \cos\left(\Delta \phi +\Delta \phi_{D}+\Delta \phi_{L}\right)\right)\\ J_{p}\left(t\right)=w\left(t\right)\cdot \left(J_{b}+J_{THz}\right) \end{array}$$

where $J_{b}$ and $J_{THz}$ are the optical bias photocurrent and induced photocurrent due to the inbound THz field, respectively. If a stable OSG is utilized, the optical bias is considered fixed. Therefore, an induced photocurrent can be estimated as the difference between the Maximal Peak Envelope (MPE) value at T=0 and T=1. Furthermore, the averaging of the induced photocurrent in the PCA detector would increase the SNR, as well as decrease the measurement uncertainty in the case of unstable OSG.

## 3. The Proposed QCW-THz System

**Figure 3:**
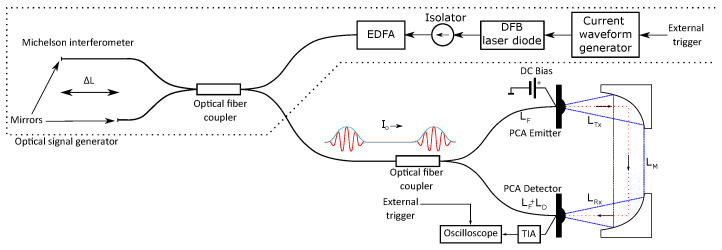
Proposed QCW-THz system’s setup.

The proposed QCW-THz system is shown in Figure 3. It uses two PCAs, for emitting and detecting THz waves and the proposed OSG. Since the optical signal is modulated, a direct current (DC) bias is used with the PCA emitter. A TIA and a high-speed oscilloscope are used to observe the induced photocurrent in the PCA detector.

### 3.1. Proposed Optical Signal Generator

The proposed OSG consists of a fast frequency swept laser source and an unbalanced optical fiber interferometer for optical signal modulation. The theoretical model of optical modulation in unbalanced optical fiber interferometers is presented in [14]. The back-reflected optical intensity from the interferometer is described as

(12) $$I_{r}\left(t\right)=\frac{I_{0}}{2}\left(1+\cos\left(\frac{2n\Delta L\omega (t)}{c}\right)\right)$$

where $I_{0}$ is the incident intensity, *n* is the effective refractive index, ΔL is the optical path difference in the optical fiber interferometer, ω(t) is the optical angular frequency, and *c* is the speed of light. If an optical frequency swept laser source is employed, the optical angular frequency becomes a function of time, and the cosine phase in Equation (12) can be estimated as

(13) $$\phi \left(t\right)=\frac{2n\Delta L}{c}\left(\omega_{0}+\frac{\partial \omega }{\partial t}t\right)$$

where $\omega_{0}$ is the initial optical angular frequency, and ∂ω/∂t is the optical angular frequency sweep rate. The back-reflected signal from the unbalanced interferometer is modulated with the frequency *f*, which can be obtained from Equation (13). If the second term in Equation (13) is set to 2π, $\omega_{0}$ is set to zero, and a fixed optical angular frequency sweep rate ∂ω/∂t is provided. The modulation frequency of the back-reflected optical intensity from an unbalanced optical fiber interferometer can be estimated as

(14) $$f=\frac{2n}{c}\Delta L\frac{\partial \nu }{\partial t}$$

where ΔL is the optical path difference (OPD), and ∂ν/∂t is the optical frequency sweep (OFS) rate. The OFS is always limited in practical laser sources. Therefore, Equation (14) can be rearranged to

(15) $$f=\frac{2n}{c}\Delta L\frac{OFSR}{T_{s}}$$

where OFSR is the optical frequency sweep range, and $T_{s}$ is the OFS duration. In the proposed optical signal generator, $T_{s}$ is equal to the driving current’s waveform duration. An overlap efficiency factor was introduced in [14], which defines the fraction of the signal subjected to the interference in the optical fiber interferometer

(16) $$u=1-\frac{n\Delta L}{c\cdot OFSR}\frac{\partial \nu }{\partial t}$$

A maximum frequency of around 1.25 THz can be achieved with the overlap efficiency factor set to u=0.5 in the proposed system. In the preliminary work [14], frequencies up to 300 GHz were achieved and confirmed with the experiments. The authors of [14] showed that the OFS rate ∂ν/∂t defines the OFS. A linear sweep and a fixed modulation frequency *f* is achieved if ∂ν/∂t is fixed.

In the proposed OSG, a compact and cost-effective tunable laser source was built using a standard Telecom DFB laser diode, driven by high and short-duration current waveforms. The fast optical frequency sweep was achieved by temperature cycling of the DFB laser diode’s active area, for which a custom current waveform generator was built. The driving current waveform for linear and fast OFS was determined experimentally [14].

Rapid temperature cycling was achieved with short and high amplitude current waveforms through the DFB laser diode’s active area. The DFB laser diode’s active area was heated up temporarily, and the DFB laser diode’s center wavelength shifted by 0.08 nm/K [18]. The temperature change in the DFB laser diode’s active area and optical frequency shift have complex relations. Current waveforms can be linear OFS or non-linear OFS [14]. In this paper, a linear OFS current waveform was used with waveform duration $T_{s}=253.46$ ns. The peak current in the proposed current waveform at $T_{s}=253.46$ ns was around 2.3 A. The DFB laser diode’s driving current is limited in duration and amplitude; therefore, the OFSR is limited as well. In the proposed fast tunable laser source utilizing a single DFB laser diode, the OFSR was limited to 1.25 THz.

**Figure 4:**
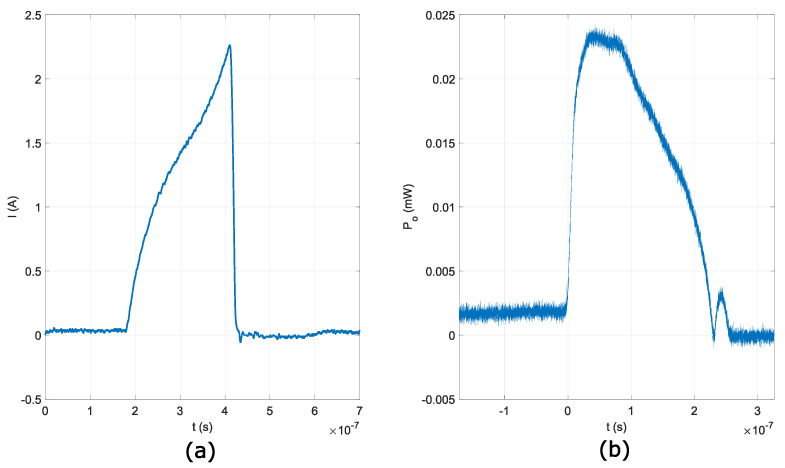
DFB laser diode ’s: (**a**) driving current waveform, (**b**) emitted optical signal.

The DFB laser diode used in the proposed OSG was not built for the proposed mode of operation. When the DFB laser diode is heated with the driving current waveform, the optical power output from the DFB laser diode starts to drop with temperature because of the thermal saturation in the DFB laser diode’s active region. Amplitude drop because of the thermal saturation in the DFB laser diode’s active area can be observed in the optical signal, as shown in Figure 4b. Before the next QCW optical signal waveform is generated, the DFB laser diode needs to cool down to the same initial temperature; therefore, the QCW signal’s repetition time needs to be smaller than the DFB laser diode’s temperature recuperation time. The maximum repetition rate for QCW signal generation is 80 kHz [19]. In the proposed system, the QCW signal’s repetition rate was set to 1.5 kHz. In addition, the DFB laser diode in the proposed OSG was cooled to −5 to ensure quicker temperature recovery of the DFB laser diode’s active area and a stable OFSR of 1.25 THz.

### 3.2. Unbalanced Optical Fiber Interferometer Design

The proposed OSG was evaluated using a Fabry–Perot and the unbalanced Michelson optical fiber interferometers in the preliminary work. In this paper, we used the unbalanced Michelson optical fiber interferometer because the back-reflected signals’ optical power can be increased. The optical fiber Michelson interferometers were built using RF-sputtering of aluminum (Al) on an optical fiber coupler’s arms. A 50% mirror reflectivity was achieved in the unbalanced manufactured optical fiber Michelson interferometers. There are still improvement possibilities with additional research on different materials with a high refractive index and manufacturing process. Nevertheless, this is not the subject of this paper, and it is not covered further. The optical fiber interferometers were designed and manufactured with an OPD of ΔL=0.03 m and ΔL=6 m. Table 1 shows the estimated target frequencies *f* based on Equation (15), where $T_{s}=253.46$ ns and OFSR=1.25 THz.

## 4. Proposed QCW-THz System’s Evaluation

The proposed QCW-THz system was evaluated in three steps: (1) Optical signal evaluation, (2) positioning of the PCA emitter and PCA detector, and (3) evaluation of THz wave detection. The QCW optical signal’s repeatability was evaluated using a fast optical detector and real-time oscilloscope. Based on Equation (7), the distance between the PCA emitter and PCA detector needs to be set according to the target frequency. In contrast, an additional optical delay needs to be added to the PCA detector’s optical signal incidence since the THz wave and optical signal should hit the PCA detector’s gap simultaneously. The additional optical delay was estimated and introduced into the proposed QCW-THz system’s setup. According to the theoretical model in Equation (11), the induced photocurrent in the PCA detector can be estimated as the difference between the induced photocurrent at T=0 and T=1; therefore, the induced photocurrent in the PCA detector was observed in two scenarios: (1) with an obstructed THz beam path, and (2) with an unobstructed THz beam path.

### 4.1. Optical Signal Evaluation

**Figure 5:**
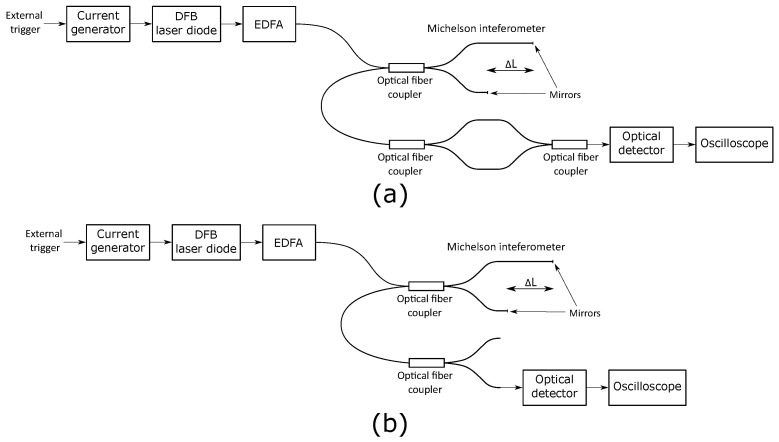
Optical signal evaluation setup for (**a**) time analysis, (**b**) output power analysis.

The generated optical signal repetition rate and maximal amplitude were evaluated using the proposed OSG and an unbalanced optical fiber interferometer for a target frequency of 1.45 GHz. The setup for time analysis is shown in Figure 5a, and the setup for performing the output power analysis is shown in Figure 5b. The setup consisted of the proposed OSG, an optical fiber coupler, a fast optical detector DXM30AF from Thorlabs, and a high-frequency oscilloscope DSA91304A from Keysight.

**Figure 6:**
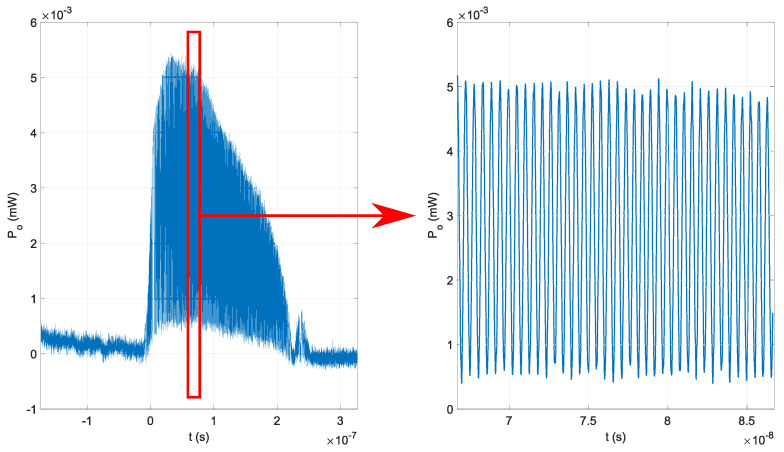
Generated optical signal without EDFA in the optical signal evaluation’s first experiment.

The first experiment was performed without the optical fiber isolator and Erbium-Doped Fiber Amplifier (EDFA). We have determined that both output signals were aligned; therefore, no additional delays were detected in the optical fibers. The result is shown in Figure 6. The maximal measured optical power without EDFA and with the Michelson interferometer with ΔL=0.03 m was 5.2 mW. The observed maximal optical power and the optical signal waveform were stable, as shown in Figure 6.

**Figure 7:**
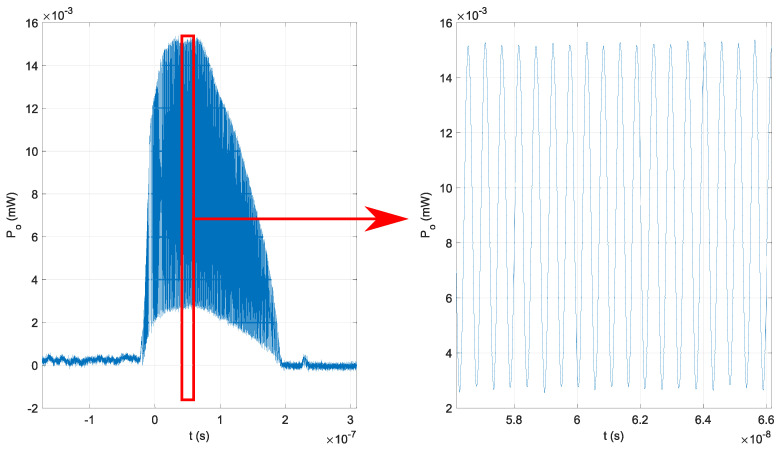
Generated optical signal with EDFA in the optical signal evaluation’s second experiment.

An EDFA, optical fiber isolator, and Michelson interferometer with ΔL=0.03 m were used in the second experiment. The EDFA, with an additional optical fiber isolator, was used between the DFB laser diode and the unbalanced optical fiber Michelson interferometer, and its output power was set to 15 mW (the nominal rating of the utilized PCAs). No additional delays or jitter were present in the generated optical signal, and the peak envelope of the optical signal was stable, as shown in Figure 7. The windowing function w(t) in (10) is now the same shape as the peak envelope of the EDFA’s output signal.

**Figure 8:**
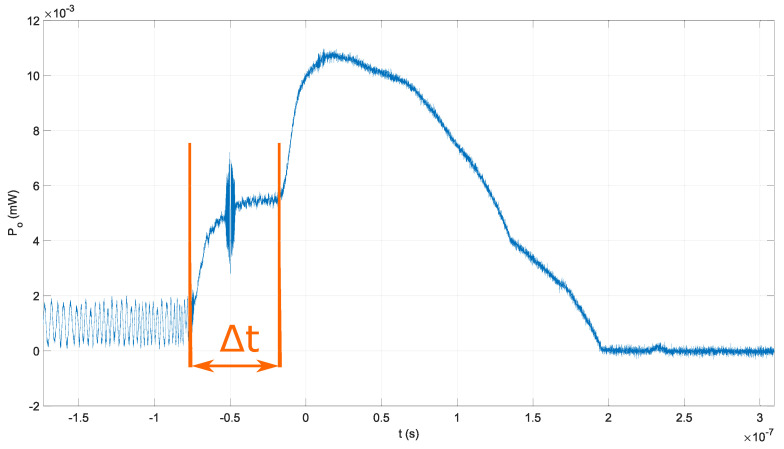
Generated optical signal with EDFA in the optical signal evaluation’s third experiment with visible delay t, which corresponds to the OPD ΔL=6 m.

In the third experiment, the EDFA, optical fiber isolator, and Michelson interferometer with ΔL=6 m were used, as shown in Figure 5b. The output power from the EDFA was set to 15 mW. The target frequency *f* in the third experiment was greater than the bandwidth of the fast optical detector. The average detected value of the optical signal in the third experiment is shown in Figure 8. Even though the average value was detected in the third experiment, the peak envelope and the average value waveform are identical. In addition, a delay Δt was introduced with the OPD ΔL=6 m in the optical fiber Michelson interferometer, as shown in Figure 8. The delay in the results from the third experiment was estimated to be Δt=59 ns, which corresponds to the optical path in optical fiber $L_{o}=\left(\Delta t\cdot c\right)/n\approx 12$ m, or OPD $\Delta L=L_{o}/2\approx 6$ m.

It can be concluded that the average detected value in the third experiment and detected peak envelope in the second experiment represent the waveform of normed windowing function w(t) in the theoretical model in Equation (10). Therefore, the induced photocurrent in the detector PCA should have an identical waveform.

### 4.2. PCA Emitter and PCA Detector Positioning

The theoretical model presented in Equation (10) shows that the positioning of the emitter and detector PCAs would impact the overall performance. Therefore, the distance between the antennas was set according to the target frequency of f=293.34 GHz, and an additional optical delay was introduced to the PCA detector’s inbound optical signal.

A PCA-FD-1550-100-TX-1 THz emitter and PCA-FD-1550-130-RX-1 THz detector from Toptica Photonics were used in the proposed QCW-THz system. Both PCAs were built on InGaAs. PCA-FD-1550-100-TX-1 is built as a p-i-n photodiode, while PCA-FD-1550-130-RX-1 is a photomixer. Both PCAs were chosen based on their bandpass, dimensions and efficiency to generate or detect THz waves. The bias voltage for the PCA emitter was set to 1 $V_{DC}$, and the PDA-S TIA from TEM Messtechnik was used to observe the induced current in the PCA detector. The detected induced photocurrent was sampled using a high-frequency oscilloscope DSA91304A from Keysight. The proposed system is shown in Figure 3.

The THz beam, emitted by the PCA emitter, was collimated using a set of collimation mirrors into the PCA detector. The distance between the two antennas had to be compensated. According to (7), the distance between two antennas needs to be a multiplier of the THz wavelength. The target frequency was f=289.33 GHz from Table 1; therefore, the distance between the antennas should be a multiplier of λ=1.022 mm. According to the user manual, the PCA emitter and PCA detector need to be placed 6 cm from the collimation mirrors. An additional distance of 5 cm was set between the collimation mirrors. The THz beam and optical signal need to interfere in the PCA detector’s gap. The THz wave’s propagation distance was compensated with an additional delay in the optical fiber, leading to the detector PCA. The delay in the optical fiber leading to the detector PCA was created by extending the length of the optical fiber. The propagation path of the THz beam is shown in Figure 3, and the propagation time $t_{p}$ was estimated using

(17) $$t_{p}=t_{TX}+t_{m}+t_{RX}$$

where $t_{TX}$ represents the THz wave’s propagation time from the PCA emitter to the collimation mirror, $t_{m}$ represents the propagation time between the collimation mirrors, and $t_{RX}$ represents the propagation time from the collimation mirror to the PCA detector. The propagation time is estimated as

(18) $$t=\frac{Ln}{c}$$

By using Equations (17) and (18), the additional length of the optical fiber leading to detector PCA was estimated as

(19) $$L_{D}=\frac{t_{p}c}{n}=11.59\;\mathrm{cm}$$

where the propagation time delay was estimated as $t_{p}=567.5$ ps. The distance between PCAs and PCAs orientation was fine-tuned using the CW-THz system TeraScan 1550 from Toptica Photonics, where the frequency was set to f=289.33 GHz. The TeraScan 1550 uses the same PCAs as were used in the proposed system. The goal of the PCAs position calibration with TeraScan 1550 was to determine the distance between the antennas and the orientation of the antennas with the maximally induced photocurrent detected. The maximal detected induced photocurrent in the off-the-shelf CW-THz system was 51 nA at 289.33 GHz.

### 4.3. Evaluation of THZ Wave Detection

The theoretical model in Equation (11) and the estimation of the induced photocurrent using the transmittance described in Section 2 were used for THz wave detection evaluation. Two different scenarios were proposed. In the first scenario, the THz beam path in the proposed QCW-THz system was blocked with a metal plate, and in the second scenario, the THz beam path in the QCW-THz system was unobstructed. The driving current waveform’s repetition rate of 5 kHz, waveform duration of T=253.46 ns and peak amplitude of 2.3 A were chosen in both scenarios. The peak optical power at the PCA detector and emitter was 15 mW, and the bias for the PCA emitter was set to 1 $V_{DC}$. The detected induced photocurrent was averaged over a 1 s time period.

**Figure 9:**
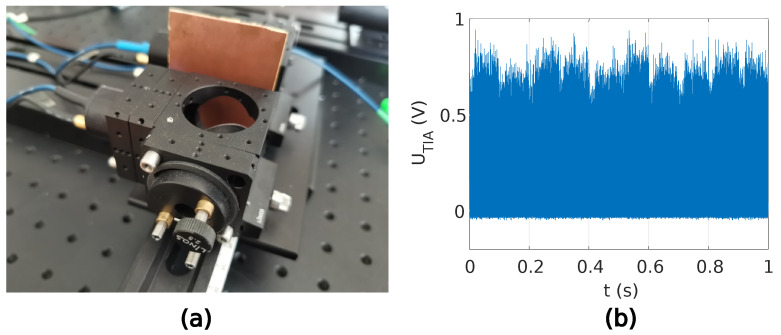
Experiment with obstructed THz beam path: (**a**) PCAs positioning with inserted copper plate, (**b**) section of the measured TIA’s output voltage.

**Figure 10:**
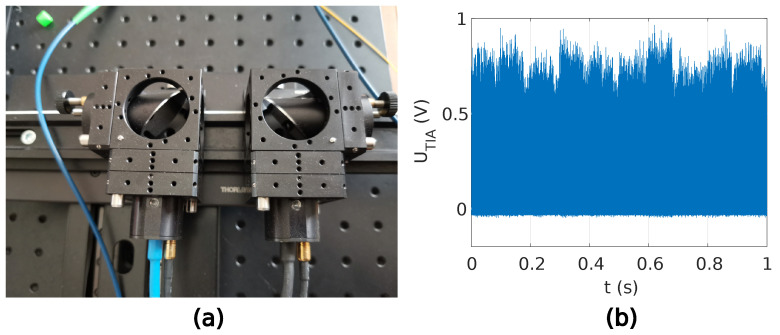
Experiment with unobstructed THz beam path: (**a**) PCAs positioning, (**b**) section of the measured TIA’s output voltage.

In the first scenario, the beam path was blocked with a metal plate, as shown in Figure 9a. It was expected that the measured induced photocurrent would consist only of optical bias; therefore, the transmittance was T=0. The average TIA voltage output was around 0.6168 V. Figure 9b shows the detected waveforms and estimated MPEs. In the second scenario, the THz beam path was unobstructed, as shown in Figure 10a. A transmittance T=1 was expected in the second scenario. The average TIA voltage output was around 0.632 V. The difference in the measured TIA voltage from the first and second scenarios represents the induced photocurrent in the PCA detector because of the inbound THz waves. With the TIA’s ratio set to $3.3\times 10^{5}$ V/A and the voltage difference between the measured TIA voltages of 0.0152 V, the induced photocurrent in the PCA detector as a result of the inbound THz filed is 46 nA.

The estimated induced photocurrent in the detector PCA was compared to the maximal induced photocurrent value obtained during the PCAs position calibration with the CW-THz system TeraScan 1550, presented in Section 4.2. The observed induced photocurrent during calibration was around 51 nA at the target frequency of 289.33 GHz. The induced photocurrent using the proposed QCW-THz system was approximately 46 nA at the same target frequency. The observed induced photocurrents are compared in Table 2. The estimated induced photocurrent in detector PCA within the proposed QCW-THz system was lower because of the currently unknown effects inside the OSG, such as polarization, dispersion, and connection losses, etc. The lower value could also be attributed to the proposed current waveform generator.

**Figure 11:**
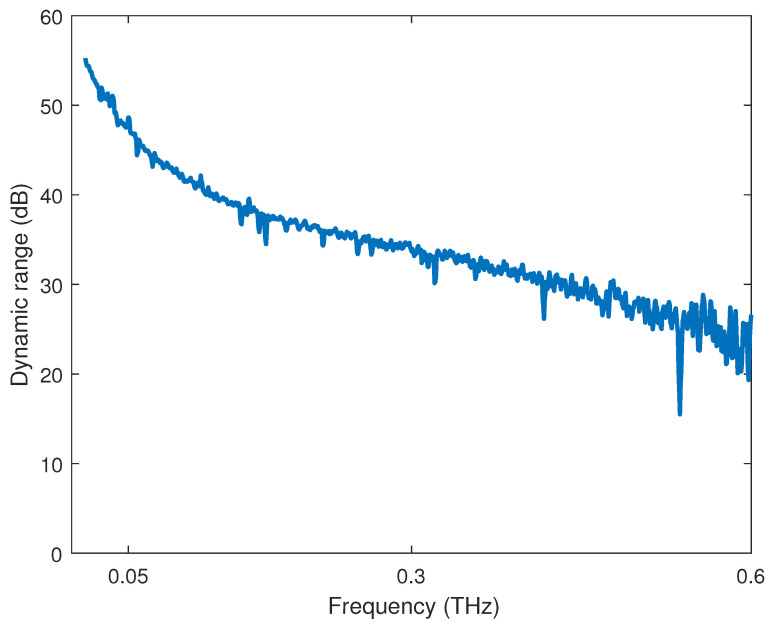
The spectral characteristic of the proposed QCW-THz system in the span of 0.1–0.6 THz with OSG resolution of 2 GHz.

The analysis of the QCW-THz has been made with two PCA antennas. The gap between the transmitter and receiver antenna is 1cm. The measured resolution of the used OSG is 2GHz in the measured frequency span of 0.1–0.6 THz. The sweep time is 80 ms. The spectral characteristic is obtained with a THz-spectrometer with 250 averages of each swept frequency and corresponds to the measured time of 20 s. Regarding the central limit theorem [20], the dynamic range is raised with the number of averages. The acquisition duration is a tradeoff between measurement speed and accuracy. The blocked THz path Figure 9a determines the noise level, where no THz information is contained in the sample, and a similar validation is presented in [21]. Figure 11 presents spectral characteristics for the proposed QCW-THz system.

**Figure 12:**
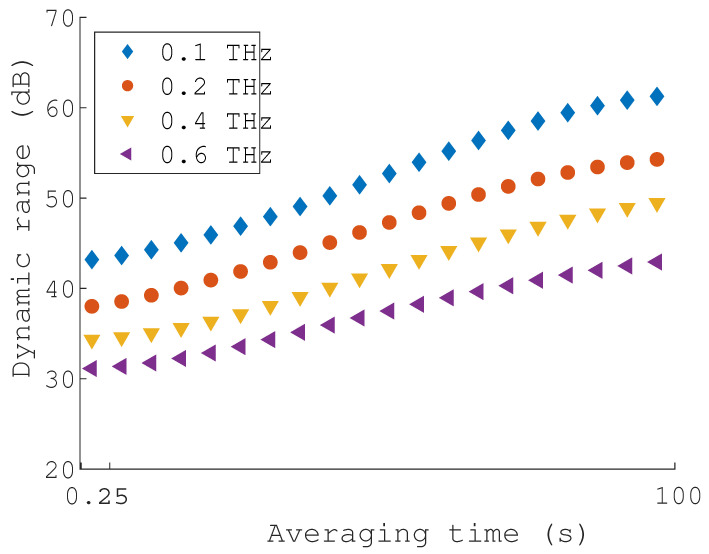
Dynamic range measurement as a function of time and averaging.

The second dynamic range measurement is evaluated as a function of the acquisition time at a specific frequency. Four different frequencies were selected [0.1, 0.2, 0.4, 0.6] THz in a time span of 0.2–100 s. For each frequency time-batch, the measurement is averaged in amplitude and phase. Figure 12 presents the dynamic range characteristic as a function of the time.

Both characteristics presented in Figure 11 and Figure 12 show the capabilities of the proposed system. The system’s dynamic range depends on the number of repetitive samples, which corresponds to the system performance and accuracy of the THz application.

## 5. Conclusions

A novel QCW-THz system is presented for generating and detecting THz waves. The proposed QCW optical signal is defined as a short burst of a CW optical signal with the THz frequency limited and normed windowing function w(t). The theoretical modeling in Section 2 has shown that the waveform of the normed windowing function w(t) remained unchanged. Furthermore, the detected induced photocurrent’s MPE at the PCA detector withheld the sample information.

The proposed QCW-THz system is based on the CW-THz system, which consists of three main parts: (1) an OSG, (2) a PCA transmitter and (3) a PCA receiver. The OSG in the proposed QCW-THz system consists of a custom and cost-effective current waveform generator, a single DFB laser diode and an optical fiber Michelson interferometer. The fast and linear OFS of the DFB laser diode was achieved by temperature cycling of the DFB’s active area with an appropriate current driving the waveform. The optical fiber Michelson interferometer was used for high-frequency modulation. The proposed OSG was evaluated and, regarding the achieved results, was deemed suitable for transmitting THz waves using a PCA emitter.

The proposed THz wave transmitter consists of a PCA emitter and bias voltage generator. In contrast, the THz receiver in the proposed system consists of a PCA detector and a TIA. The theoretical model showed that the distance between the PCA emitter and detector introduces an additional phase delay. The phase delay due to the distance between the PCAs was compensated in the proposed system using an optical delay line at the PCA detector. Furthermore, the theoretical model showed that the MPE of the detected induced photocurrent in the PCA detector withholds the sample information. The evaluation experiment was designed based on the transmittance evaluation. The reference MPE value was acquired while the THz beam path was unobstructed (T=1), and the reference MPE value was compared to the MPE value obtained with the blocked THz beam path with a metal plate (T=0). The difference between the measured MPE values represents the induced photocurrent due to the inbound THz field. The principal operation of the proposed QCW-THz system is confirmed in this paper. The main drawback is that the MPE value was averaged over the time span of 1s, and the observed MPE varied throughout the measurement. The MPE variation is a result of the proposed method itself. The temperature cycling of the DFB laser diode needs to be exact in terms of temperature recuperation. If it is not, the OFS range will change, and the modulation frequency will be uncertain. The THz wave’s phase can change with varying the modulation frequency, and the developed theoretical model showed that a change in the THz wave’s phase would change the amplitude.

The proposed system is limited mainly by the optical signal generator. With a slightly non-linear OFS, the back-reflected optical signal from the unbalanced optical fiber Michelson interferometer will have a broader spectral line. Later, it would cause a signal distortion in both the PCA emitter and detector. The effects of the proposed OSG are relatively unknown. They require additional research to achieve optimal operation and extended tunability of the proposed OSG.

The QCW-THz system proposed in this paper could also be classified as a broadband system. Therefore, similar techniques for extracting phase information could be used in future development. The optical delay is swept in the THz broadband system. The delay at the maximal detected THz power represents the phase delay. In the proposed system, the distance between the antennas could serve as a delay line. Phase information could be extracted by sweeping the distance between the antenna in the proposed system.

## Figures and Tables

**Table 1:** Estimated target frequencies at OPD ΔL=0.03 m and ΔL=6 m.

OPD, ΔL	0.03 m	6 m
**Estimated Frequency, *f***	1.45 GHz	289.33 GHz

**Table 2:** Comparison between the observed photocurrent at detector PCA using the off-the-shelf CW-THz system and the proposed system at the same target frequency f=289.33 GHz.

Platform	TeraScan 1550	Proposed QCW-THz System
**Observed induced photocurrent**	51 nA	46 nA
